# Supplementary material for: The high expression of MTH1 and NUDT5 predict a poor survival and are associated with malignancy of esophageal squamous cell carcinoma
Source: PeerJ. 2020 May 26;8:e9195. doi: 10.7717/peerj.9195 (PMC7258951; doi:10.7717/peerj.9195)

Figure 1 A-H

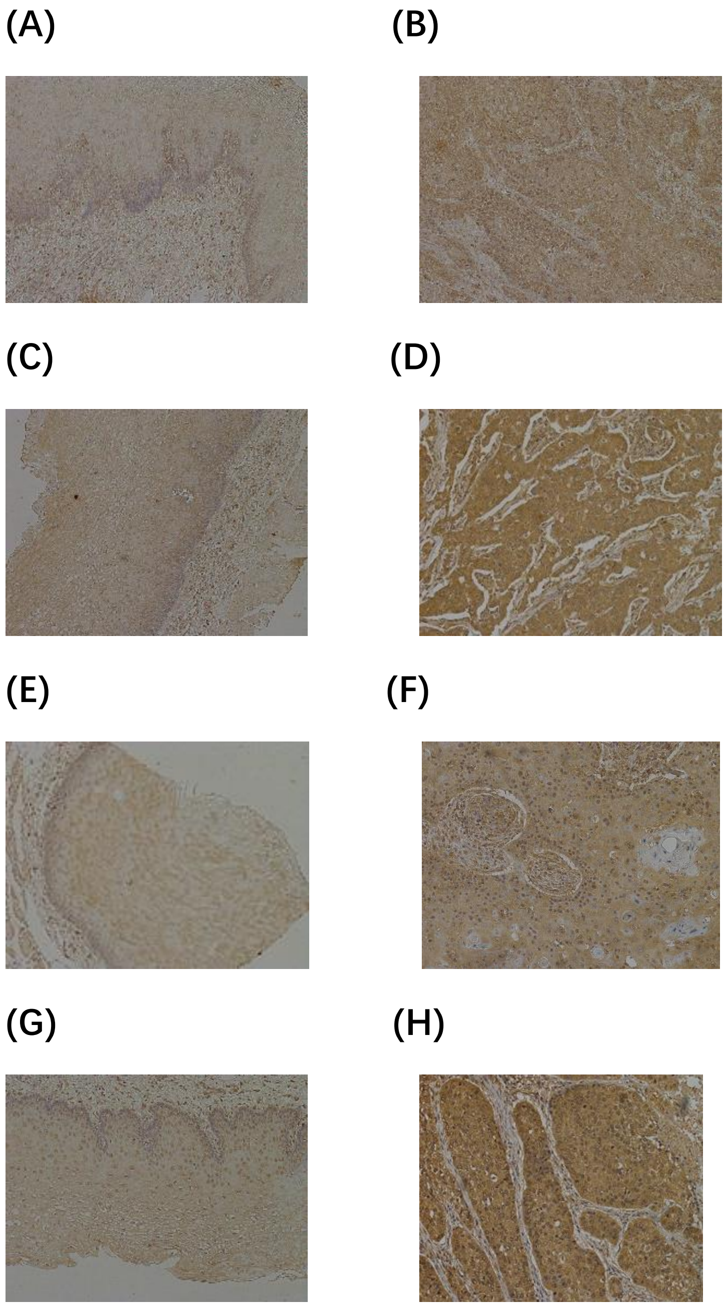

Figure 2A

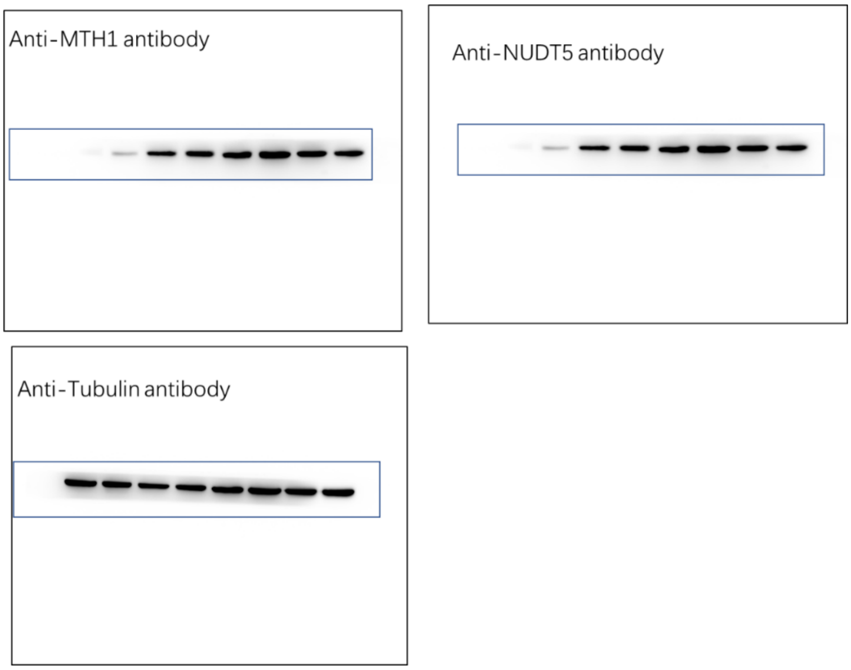

Figure 2D

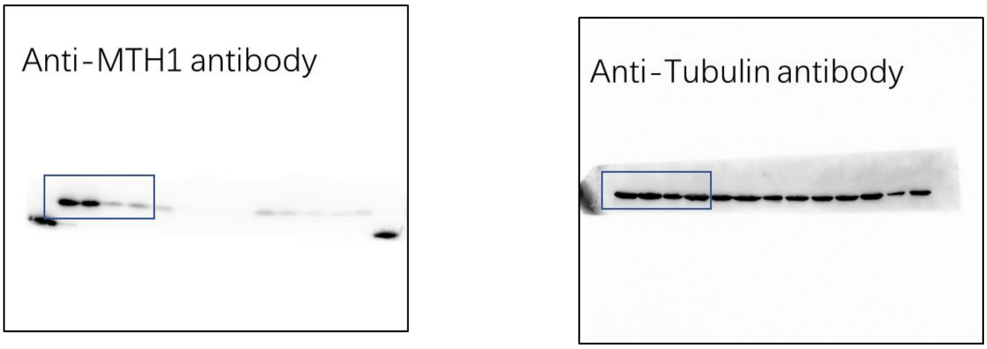

Figure 2E

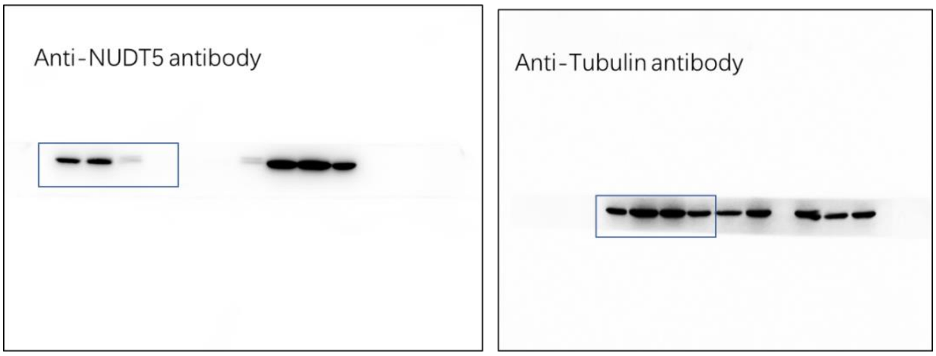

Figure 3M

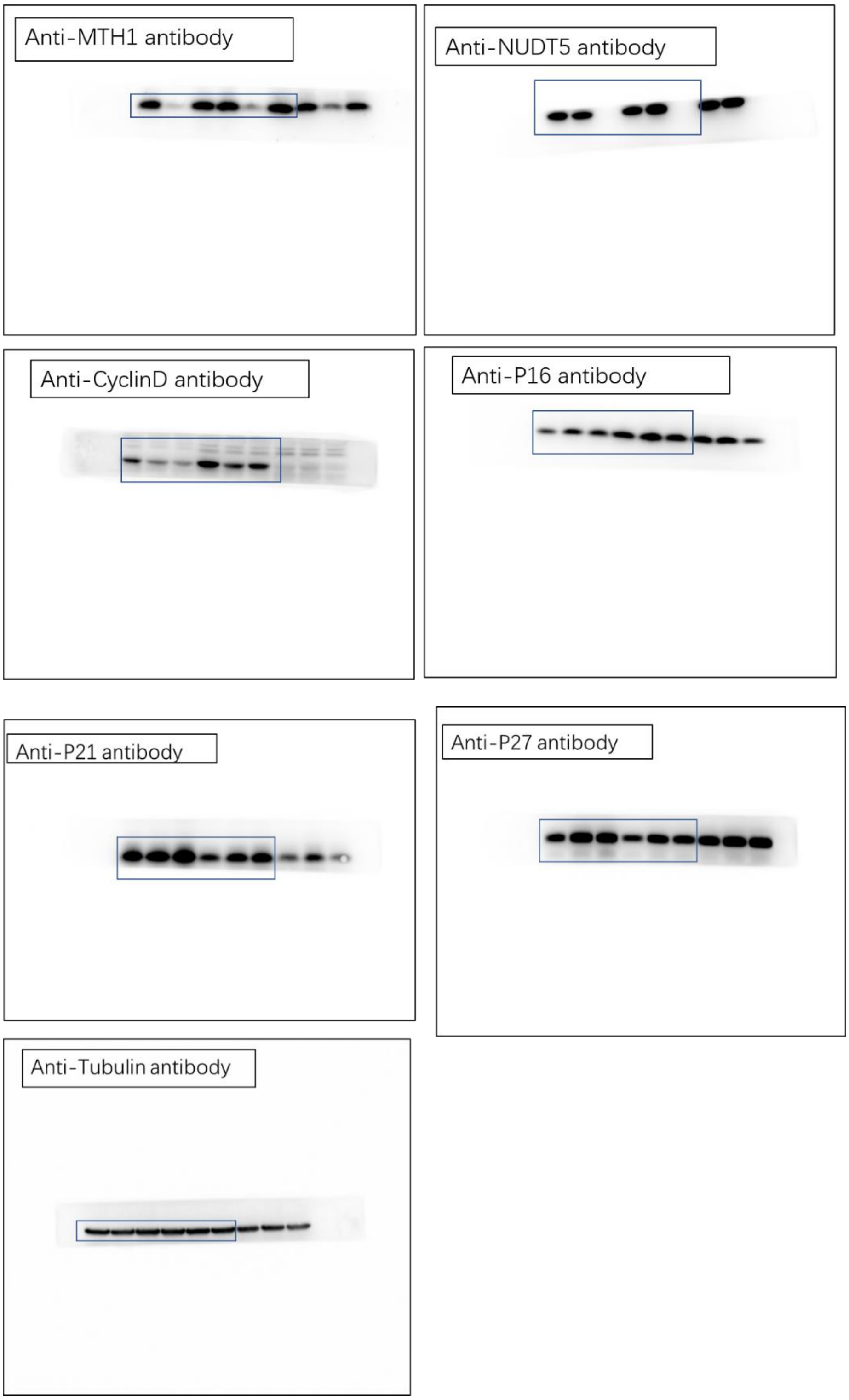

Anti-MTH1 antibody
Anti-NUDT5 antibody
Anti-CyclinD antibody
Anti-P16 antibody
Anti-P21 antibody
Anti-P27 antibody
Anti-Tubulin antibody

Figure 4Q

Repeat 1

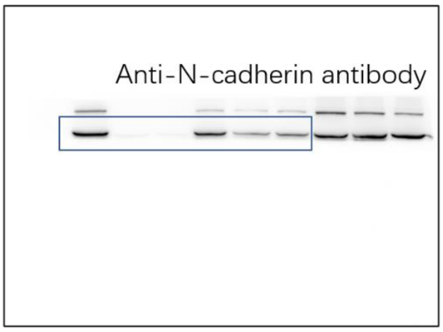

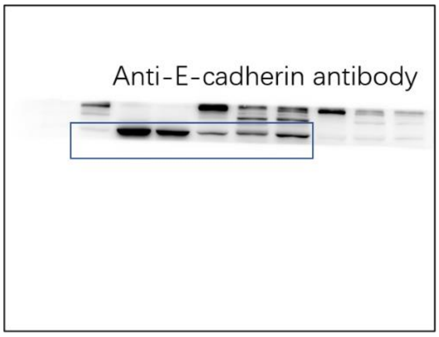

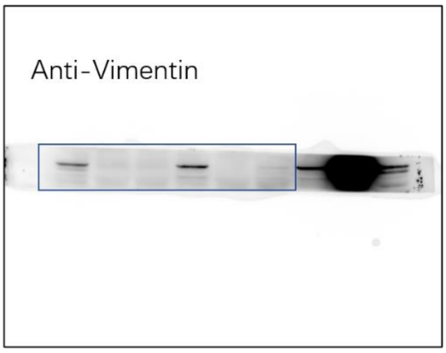

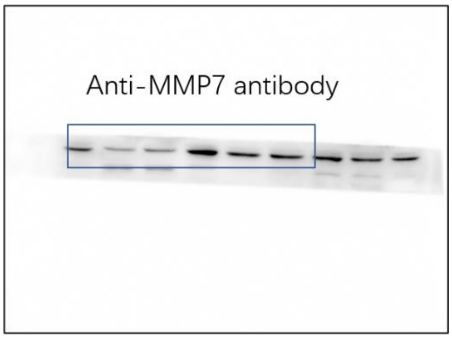

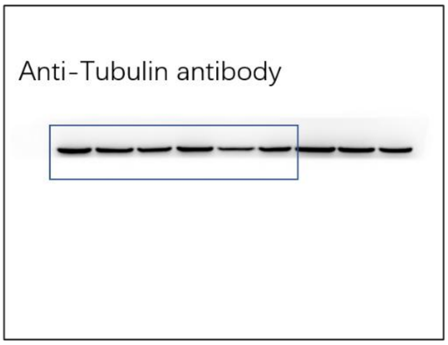

Repeat 2

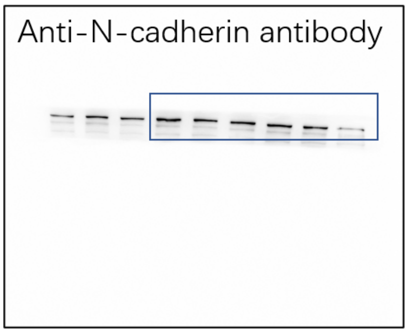
Anti-N-cadherin antibody

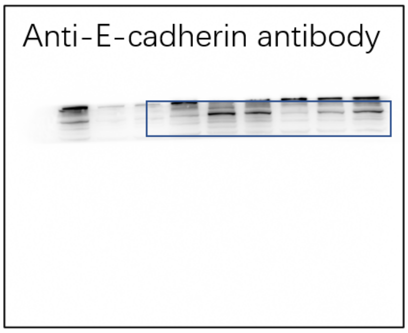
Anti-E-cadherin antibody

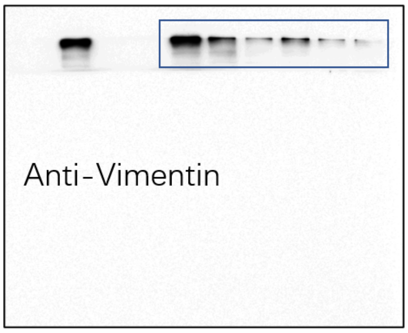
Anti-Vimentin

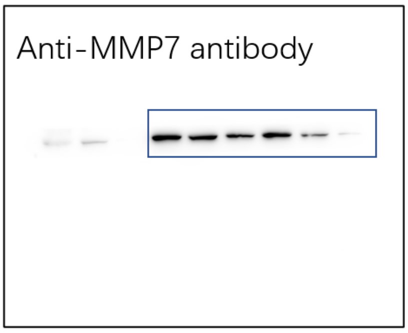
Anti-MMP7 antibody

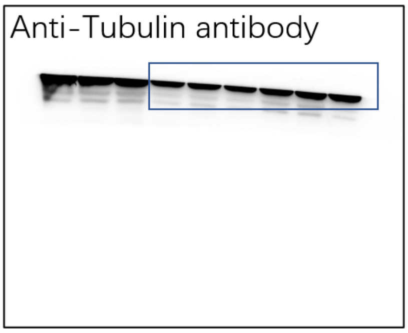
Anti-Tubulin antibody

Repeat 3

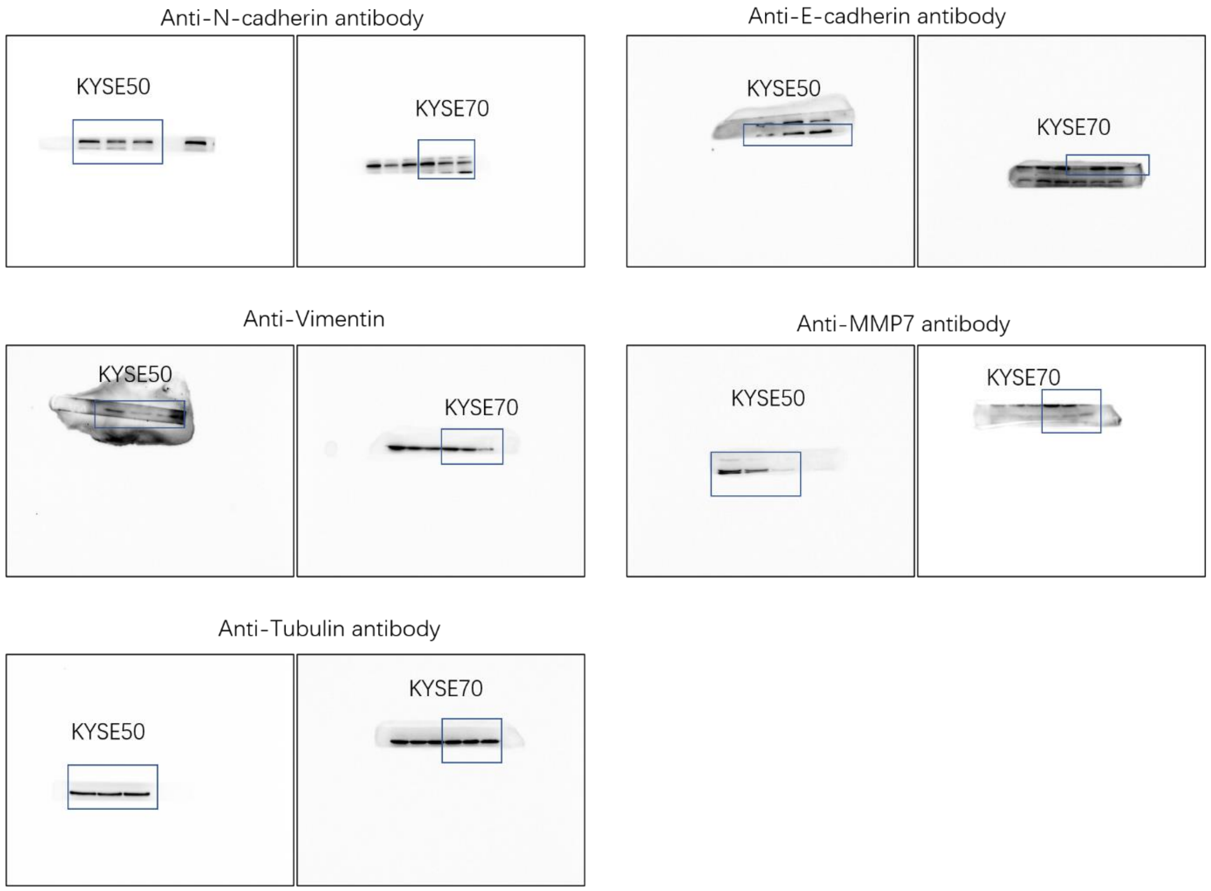
Anti-N-cadherin antibody
KYSE50
KYSE70
Anti-E-cadherin antibody
KYSE50
KYSE70
Anti-Vimentin
KYSE50
KYSE70
Anti-MMP7 antibody
KYSE50
KYSE70
Anti-Tubulin antibody
KYSE50
KYSE70

Figure 4R

Repeat 1

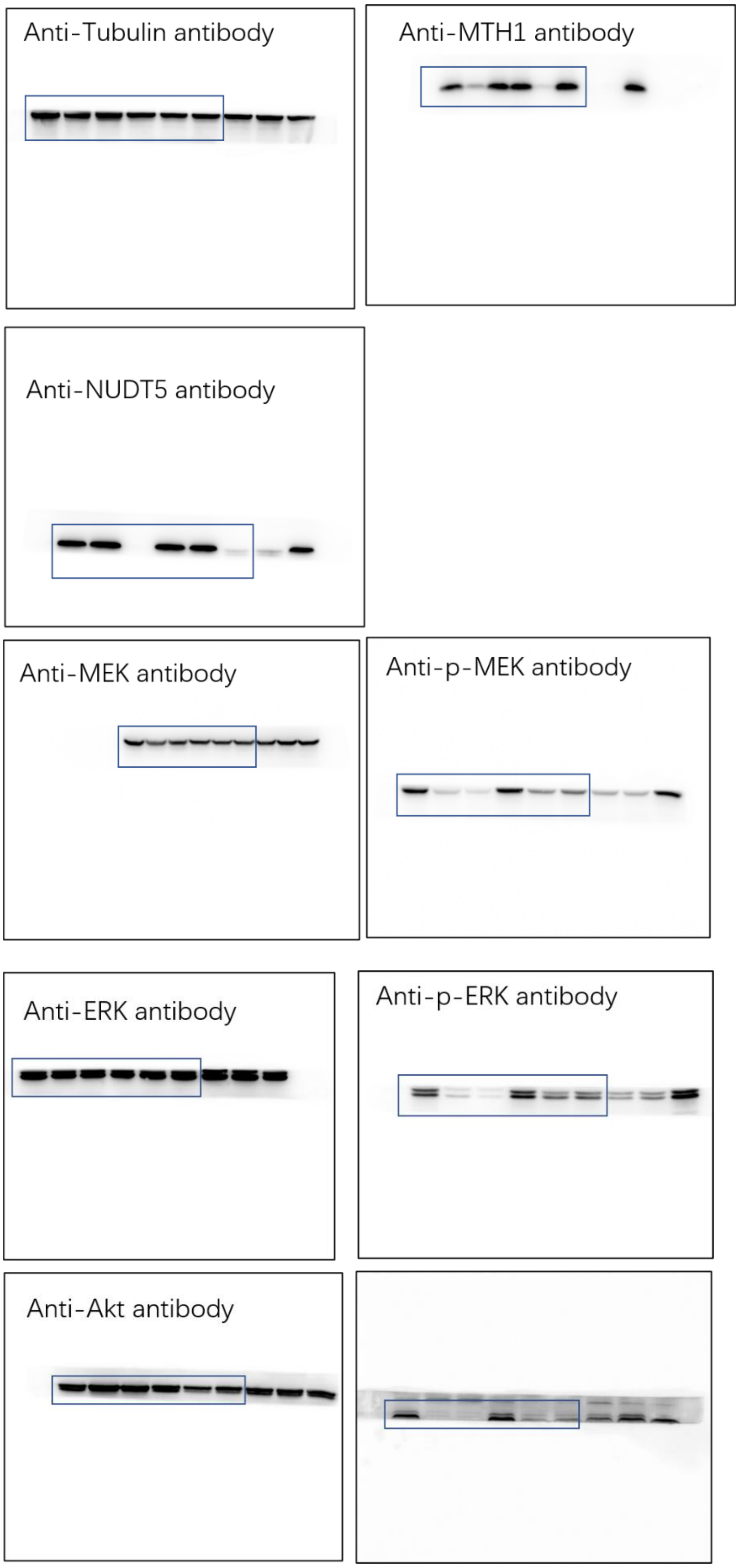

Repeat 2

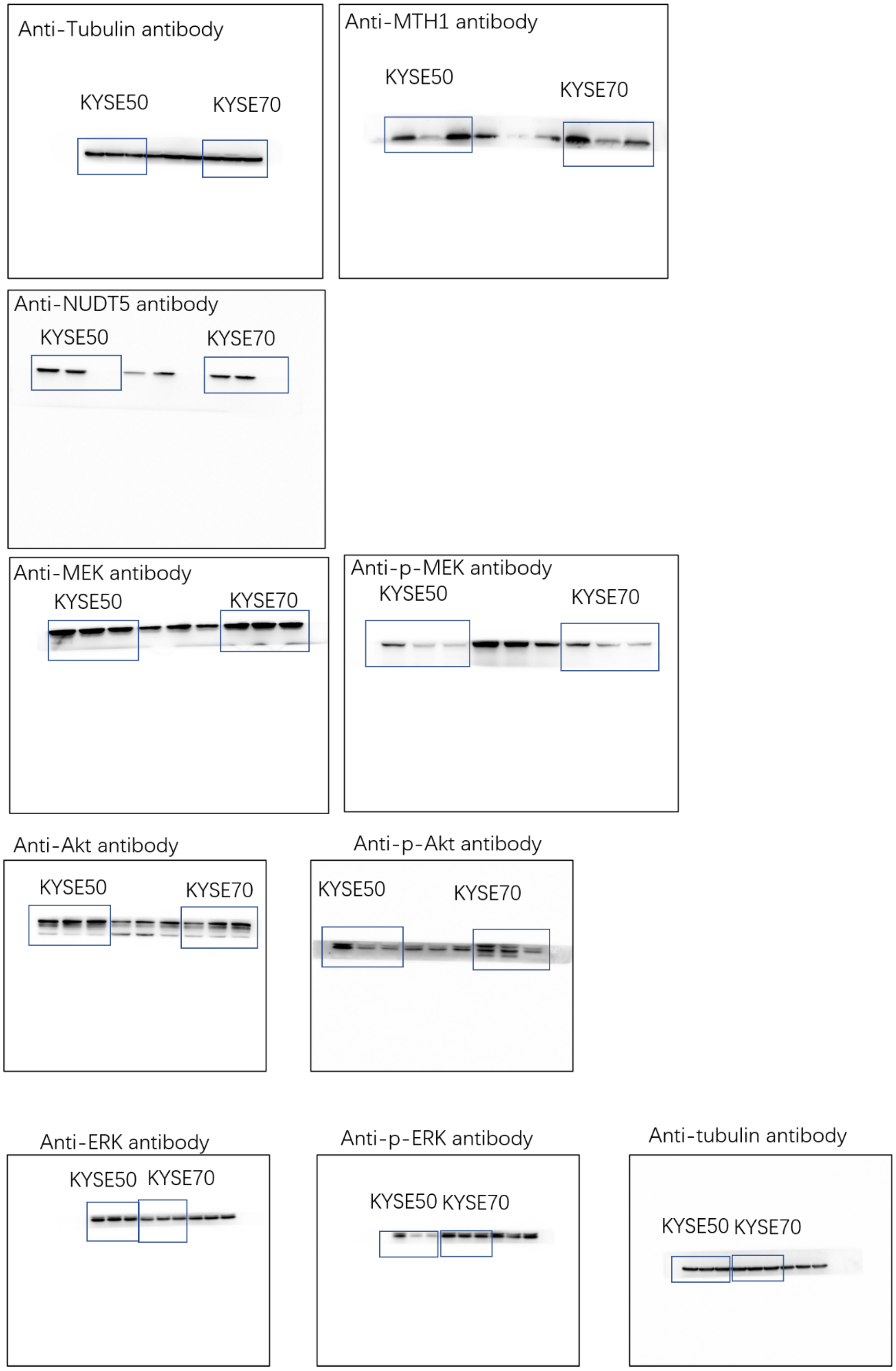
Anti-Tubulin antibody
KYSE50
KYSE70
Anti-MTH1 antibody
KYSE50
KYSE70
Anti-NUDT5 antibody
KYSE50
KYSE70
Anti-MEK antibody
KYSE50
KYSE70
Anti-p-MEK antibody
KYSE50
KYSE70
Anti-Akt antibody
KYSE50
KYSE70
Anti-p-Akt antibody
KYSE50
KYSE70
Anti-ERK antibody
KYSE50
KYSE70
Anti-p-ERK antibody
KYSE50
KYSE70
Anti-tubulin antibody
KYSE50
KYSE70

Repeat 3

Anti-Tubulin antibody

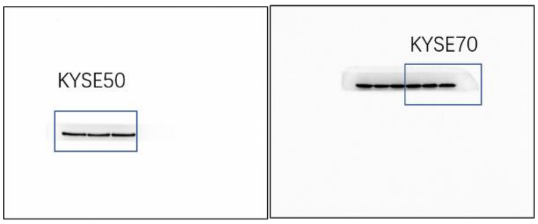

Anti-MTH1 antibody

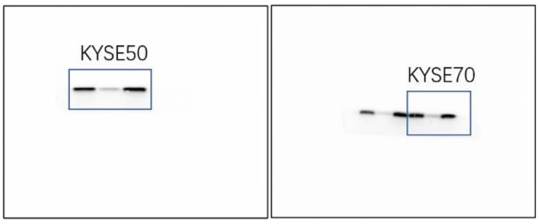

Anti-NUDT5 antibody

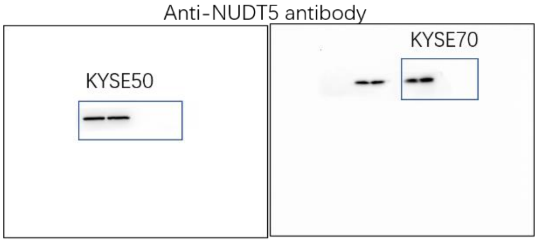

Anti-MEK antibody

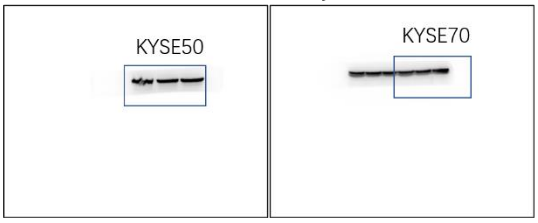

Anti-p-MEK antibody

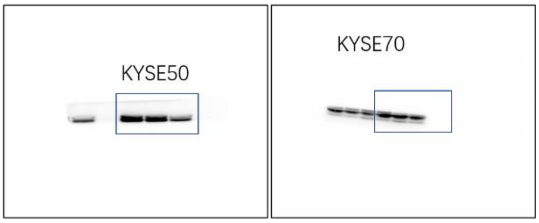

Anti-ERK antibody

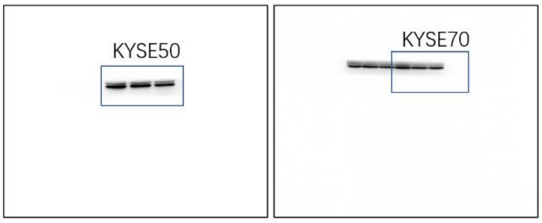

Anti-p-ERK antibody

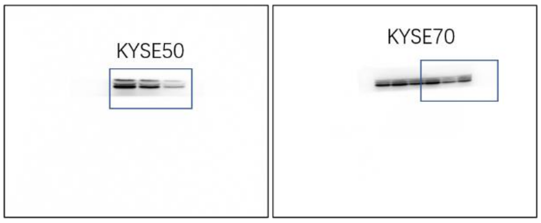

Anti-Akt antibody

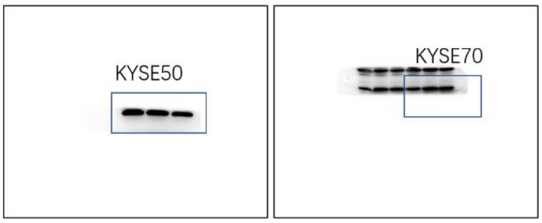

Anti-p-Akt antibody

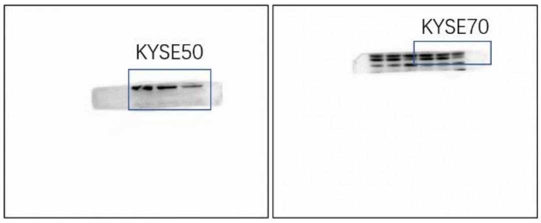

Supplement: Supplemental Information 2 [file peerj-08-9195-s002.pdf]
